# Plastid genome comparison and phylogenetic analyses of the Chinese group of medicinal species and related taxa within *Asparagus* genus

**DOI:** 10.3389/fpls.2025.1508898

**Published:** 2025-01-27

**Authors:** Pingxuan Xie, Tinglu Wang, Jiawei Tan, Linyuan Fan, Changkun Liu, Hanjing Yan

**Affiliations:** ^1^ School of Traditional Chinese Medicine, Guangdong Pharmaceutical University, Guangzhou, China; ^2^ Yunnan General Administration of Forestry Seeds and Seedlings, Kunming, China; ^3^ College of Resources Environment and Chemistry, Chuxiong Normal University, Chuxiong, China

**Keywords:** *Asparagus*, plastid genome, simple sequence repeats, divergent hotspots, phylogenetic relationships, species identification

## Abstract

**Background:**

*Asparagus* L. is a large genus widely distributed across the continents of the Old World. Among its members, approximately 14 species found in China are recognized as popular herbal medicines. However, accurate authentication of these medicinal species and their phylogenetic relationships with related taxa remains unresolved.

**Methods:**

To identify simple sequence repeats (SSRs) and divergence hotspot regions appropriate for future authentication studies, as well as to infer the phylogenetic relationships among *Asparagus* species, we employed a plastid genome (plastome) dataset consisting of 25 *Asparagus* species (21 newly sequenced and four retrieved from GenBank), encompassing 12 Chinese medicinal species, for comparative and phylogenetic analyses.

**Results:**

All *Asparagus* plastomes displayed a typical quadripartite structure with sizes ranging from 155,948 bp to 157,128 bp and harbored 114 unique genes (80 protein-coding genes, 30 tRNA genes, and four rRNA genes). IRscope and Mauve analyses indicated minimal structural variation among *Asparagus* plastomes. We detected between 79 to 95 SSRs across the plastomes; most were located in the large single-copy (LSC) region and primarily consisted of mono-nucleotide repeat sequences (especially A and T repeats). The genus displayed mono-, di-, tri-, tetra-, penta-, and hexa-nucleotide repeats, but with variations in types and numbers among different species. Additionally, we identified 12 special SSR motifs and seven divergent hotspot regions that may serve as potential molecular markers for future identification efforts. Phylogenetic analyses yielded a robust phylogeny for *Asparagus* taxa, which were split into Clades I, II, and III. Notably, medicinal *Asparagus* species were mainly found in Clade III. Although the phylogenetic relationships of most *Asparagus* species aligned with previous study findings, the phylogenetic positions of *A. munitus*, *A. subscandens*, *A. gobicus*, and *A. dauricus* were newly determined.

**Conclusions:**

The plastomes of *Asparagus* are largely conserved in terms of genome structure, size, gene content, and arrangement. Nevertheless, SSRs analyses revealed significant interspecific polymorphism within *Asparagus*. In addition, special SSR motifs and divergent hotspot regions identified from *Asparagus* plastomes provided reference for subsequent identification investigations. The plastome-based phylogeny provided preliminary insights into the relationships among the Chinese group of medicinal species and related taxa within *Asparagus*. Overall, this study offers a wealth of informative genetic resources pertinent to *Asparagus*, thereby enhancing our understanding of its evolution and laying a foundation for species identification, assessment of genetic population diversity, as well as the exploration and conservation of germplasm resources.

## Introduction

*Asparagus* L. (Asparagaceae), a genus comprising herbaceous and sub-shrubby members, as well as scramblers and climbers, encompasses 170 to 300 species that are widely distributed across the continents of the Old World (with only northern Australia in the Antipodes) (Stevens, 2024). Many of these species are known for their edible, ornamental, or medicinal applications (Amel et al., 2020). Currently, 31 *Asparagus* species (15 endemic, two introduced) have been documented in China (Chen and Tamanian, 2000), with approximately 14 species recognized for their significant medicinal value as herbal medicines (Editorial Committee of Chinese Materia Medica of the National Administration of Traditional Chinese Medicine, 1999; Yunnan Food and Drug Administration, 2005; Sichuan Food and Drug Administration, 2010). Tiandong, a well-known traditional Chinese medicine listed in the Chinese Pharmacopoeia, is derived from the dried root tubers of *A. cochinchinensis* (Lour.) Merr., which is effective in treating a wide range of ailments, including asthma, cough, constipation, thrombosis, and inflammatory diseases (Chinese Pharmacopoeia Commission, 2020; Wang et al., 2022a). Other medicinal *Asparagus* species, including *A. filicinus* Buch.-Ham. ex D.Don, *A. meioclados* H.Lév., *A. gobicus* Ivanova ex Grubov, *A. subscandens* F.T.Wang & S.C.Chen, *A. brachyphyllus* Turcz., *A. lycopodineus* (Baker) F.T.Wang & Tang, *A. officinalis* L., *A. setaceus* (Kunth) Jessop, *A. racemosus* Willd., *A. myriacanthus* F.T.Wang & S.C.Chen, *A. kansuensis* F.T. Wang & Tang, *A. munitus* F.T.Wang & S.C.Chen, and *A. taliensis* F.T.Wang & Tang ex S.C.Chen, are utilized as herbal medicines in traditional remedies; some of these exhibit effects similar to those of *A. cochinchinensis* or possess unique therapeutic efficacy (Editorial Committee of Chinese Materia Medica of the National Administration of Traditional Chinese Medicine, 1999; Yunnan Food and Drug Administration, 2005; Sichuan Food and Drug Administration, 2010; Lv and Chen, 2019; Parihar and Sharma, 2021; Li and Huang, 2021).

Nevertheless, the accurate authentication of *A. cochinchinensis* and other congeneric species continues to pose significant challenges. The abundant interspecific and even intraspecific morphological variations complicate species identification (Afroz et al., 2022). Moreover, the overlapping phenotypic traits of species within the genus to varying degrees often lead to confusion or admixture among congeneric species in practical applications. In certain regions of China, *Asparagus* species—particularly those with similar swollen roots, such as *A. filicinus*, *A. meioclados*, *A. munitus*, *A. subscandens*, *A. taliensis*, *A. brachyphyllus*, and *A. lycopodineus*—are frequently misidentified as *A. cochinchinensis* (Editorial Committee of Chinese Materia Medica of the National Administration of Traditional Chinese Medicine, 1999; Yunnan Food and Drug Administration, 2005; Sichuan Food and Drug Administration, 2010; Chen et al., 2000; Lv and Chen, 2019), undermining the quality control of Tiandong and posing potential risks to drug safety. Although several studies have aimed to differentiate medicinal species from other congeneric taxa, these efforts have not comprehensively addressed the issue due to inadequate molecular data (*trnH-psbA*, *trnL-F*) or insufficient sampling of medicinal species (Huang et al., 2010; Ou et al., 2010, Ou et al., 2013).

Furthermore, the evolutionary relationships between these medicinal species and related taxa of *Asparagus* distributed in China remain inadequately defined. Previous studies on this genus were generally taxonomic and did not specifically take into account the explicit phylogeny (Fukuda et al., 2005; Kubota et al., 2012; Norup et al., 2015). Meanwhile, the sparse or inadequate sampling has resulted in a poor understanding of interspecific relationships among *Asparagus* species (Sheng, 2022; Wong et al., 2022; Tian et al., 2023). Recently, Bentz et al. (2024) reconstructed the phylogeny of *Asparagus* based on a majority of plastid protein-coding genes. Despite including many Chinese *Asparagus* species, the taxonomic sampling of medicinal species still requires improvement. Additionally, excluding *ycf1*, *ycf2*, *accD*, and *infA* genes from phylogenetic analysis, as well as the lack of non-coding regions of plastid DNA commonly used in closely related species phylogenetic studies (Borsch et al., 2009), may result in some discrepancies in interspecific relationships. Therefore, to accurately identify species and further investigate the phylogenetic relationships between Chinese *Asparagus* medicinal species and related taxa, a broader sampling of medicinal species along with additional genomic data would be beneficial.

Despite being relatively conservative (Ravi et al., 2008; Jansen and Ruhlman, 2012), plastomes with abundant genetic variants have been widely recognized as a powerful tool for phylogenetic reconstruction across various taxonomic levels (Gitzendanner et al., 2018; Givnish et al., 2018; Wu et al., 2021; Ji et al., 2023; Ma et al., 2014; Guo et al., 2022). Meanwhile, the characteristics and structural patterns of the plastome obtained through comparative analysis also provide supplementary information for phylogenetic inference (Liu et al., 2018; Lyko and Wicke, 2021). Additionally, divergence hotspots and simple sequence repeats (SSRs) derived from plastome data may serve as candidate DNA barcodes or efficient molecular markers for species identification (Downie and Jansen, 2015; Zhou et al., 2023).

In this study, a dataset comprising 25 complete *Asparagus* plastomes (21 newly sequenced and four obtained from GenBank) was utilized for comparative and phylogenetic analyses, with 12 out of 14 *Asparagus* medicinal species included. Our primary objectives are to (1) investigate the plastome structure and sequence divergence of *Asparagus* plants; (2) identify SSRs and divergence hotspots of *Asparagus*; and (3) preliminarily elucidate the phylogenetic relationships among the Chinese group of medicinal species and related taxa within *Asparagus*.

## Materials and methods

### Plant sampling, DNA extraction and sequencing

Fresh leaves from 21 *Asparagus* species were collected and dried with silica gel. The identifications of the samples were conducted by Professor Heng Li. Voucher specimens were deposited in the Herbarium of Kunming Institute of Botany, CAS (KUN), Chinese Academy of Sciences, Yunnan, China, with collection details provided in **Supplementary Table 1**. Plastid genomic DNA was extracted from approximately 5 mg of silica gel-dried leaf tissues using the CTAB method (Doyle and Doyle, 1987). Total DNA was fragmented into 400 bp for pair-end library preparation utilizing a TruSeq DNA Sample Prep Kit (Illumina, Inc., San Diego, CA, USA) according to the manufacturer’s instructions. All genomic data were sequenced on the Illumina NovaSeq platform at Personalbio (Shanghai, China).

### Plastome assembling and annotation

After filtering the raw data using fastP v0.15.0 (Chen et al., 2018) with parameters -n 10 and -q 15, high-quality reads were obtained. Subsequently, these high-quality reads were employed to assemble plastome through NOVOPlasty v2.6.2 (Dierckxsens et al., 2017) with the default settings, employing the *rbcL* sequence from *A. officinalis* (JQ273904) as a seed reference. The plastomes were annotated using the CPGAVAS2 (Shi et al., 2019) and further checked manually using Geneious v10.2.3 (Kearse et al., 2012). Additionally, the circular maps of the plastomes were generated using OrganellarGenomeDRAW (OGDRAW) (Greiner et al., 2019). GenBank accession numbers for the 21 *Asparagus* plastomes were documented in **Supplementary Table 1**. Together with six available plastomes obtained from NCBI (**Supplementary Table 2**), a total of 25 plastomes from *Asparagus* and two outgroup species were included in follow-up analyses.

### Simple sequence repeat analysis

Simple sequence repeats (SSRs) were identified in each *Asparagus* plastome by using MISA (http://pgrc.ipk-gatersleben.de/misa/), with the thresholds for repeat units set at ten, five, four, three, three, and three, for mono-, di-, tri-, tetra-, penta- and hexa-nucleotide repeats, respectively.

### Genomic comparison and divergence hotspots analysis

To investigate the structural variation of plastomes in *Asparagus*, we compared the expansion and contraction of inverted repeat (IR) regions across these plastomes using IRscope (Amiryousefi et al., 2018). After the removal of one copy of the IR region, the general structural characteristics of *Asparagus* plastome were further analyzed with Mauve v2.3.1 (Darling et al., 2004). Furthermore, the sequence divergence of *Asparagus* plastomes was assessed using the mVISTA (Frazer et al., 2004) in the Shuffle-LAGAN alignment program, with the plastome of *A. cochinchinensis* set as the reference. The nucleotide variability values (π) for the alignment of *Asparagus* plastomes were calculated using DnaSP v.5.0 (Librado and Rozas, 2009), with the window length and step size set to 600 bp and 200 bp, respectively. Coding and non-coding regions with π ≥ 0.010 were extracted using Geneious v10.2.3 (Kearse et al., 2012) and their number of variable sites and parsimony-informative (parsim-info) sites were evaluated by MEGA X (Kumar et al., 2018).

### Phylogenetic analyses

To infer the phylogenetic relationships among *Asparagus* species, phylogenomic analyses were conducted to reconstruct phylogenetic trees based on 27 plastomes (**Supplementary Table 1**; **Supplementary Table 2**). *Cordyline indivisa* (G. Forst.) Endl. and *Eustrephus latifolius* R.Br. were utilized as outgroup species with the findings of Ji et al. (2023). All plastomes were aligned using MAFFT v7.450 (Katoh and Standley, 2013) with the default settings, and then manually adjusted in Geneious v10.2.3 (Amiryousefi et al., 2018). The characteristics of plastome alignments were analyzed using MEGA X (Kumar et al., 2018). Maximum likelihood (ML) analysis was conducted using the RAxML-HPC2 tool on XSEDE within the CIPRES Science Gateway platform (http://www.phylo.org/portal2), under a GTR + GAMMA model along with 1000 rapid bootstrap replicates. Bayesian Inference (BI) was executed by utilizing MrBayes v3.2.7a (Ronquist et al., 2012) with the most suitable substitution model (GTR + I + G) chosen through Modeltest v3.7 (Posada and Crandall, 1998). The independent run of Markov Chain Monte Carlo (MCMC) was conducted for 1,000,000 generations, with sampling performed every 100 generations and the first 25% of generations disregarded as burn-in for each matrix. Stationarity was attained when the average standard deviation of split frequencies fell below 0.01. The software FigTree v1.4.2 (Rambaut, 2014) was employed to visualize and modify the phylogenetic trees.

## Results

### Characteristics of the *Asparagus* plastomes

**Figure 1:**
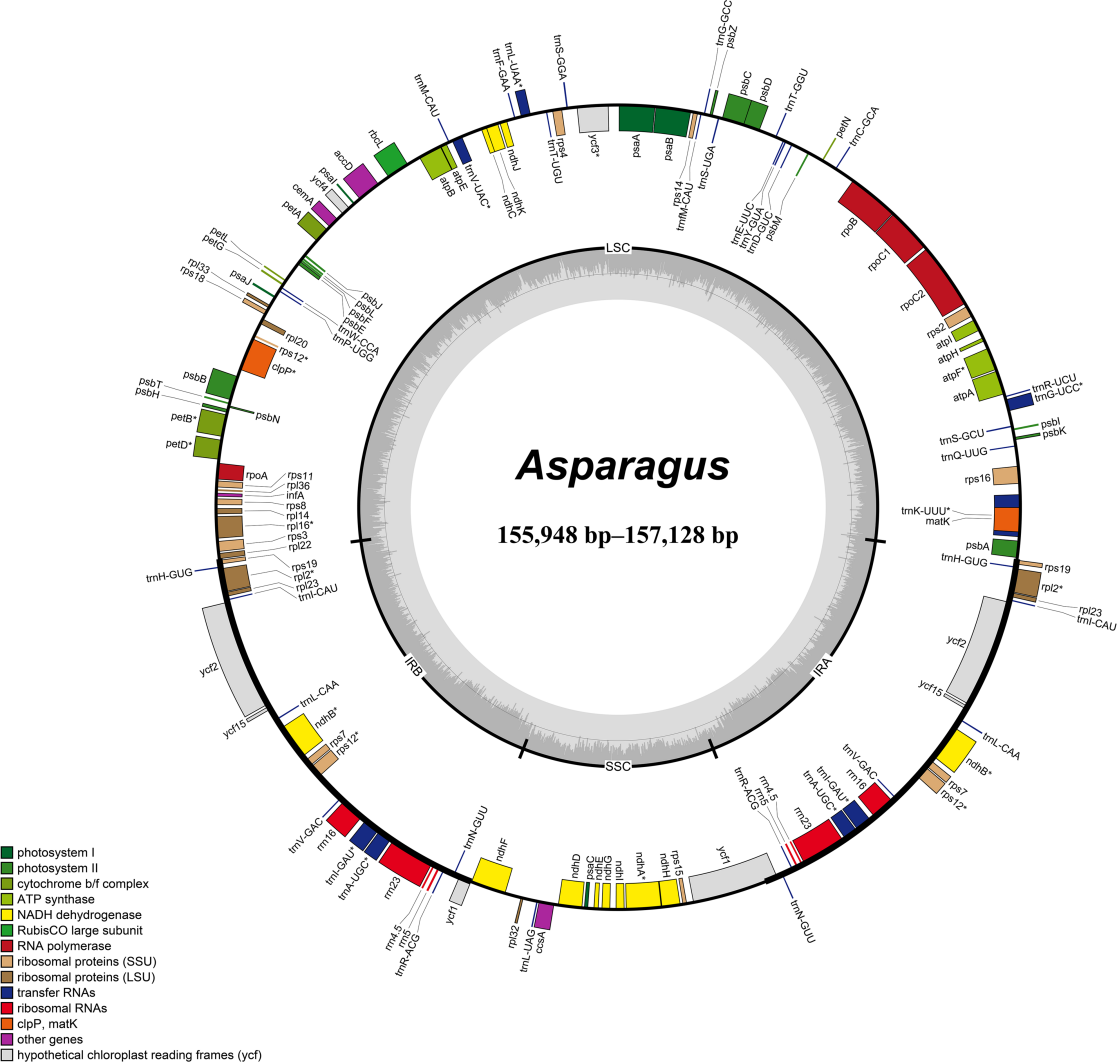
Circular maps of *Asparagus* plastomes. The inside and outside of the circle are genes transcribed clockwise and counterclockwise, respectively. The genes belonging to different functional groups are color-coded. The dashed area in the inner circle shows GC content.

The summary of the low-coverage genome sequencing and assembly of plastomes is presented in **Supplementary Table 3**. The plastomes of *Asparagus* species exhibited sizes ranging from 155,948 bp (*A. taliensis*) to 157,128 bp (*A. falcatus* L.), which displayed a typical quadripartite structure, comprising two inverted repeat regions (IRs, 26,441–26,573 bp), a large single-copy (LSC) region (84,196–85,343 bp), and a small single-copy (SSC) region (18,621–18,732 bp) (**Table 1**; **Figure 1**). The total GC content of the plastomes varied between 37.5% to 37.6% (**Table 1**). Each plastome harbored 114 unique genes, including 80 protein-coding genes, 30 tRNA genes, and four rRNA genes (**Table 1**; **Supplementary Table 4**). Among them, the *ycf15* gene was annotated as a pseudogene.

**Table 1:** Comparison of plastome features among *Asparagus* plants.

Species	Size (bp)				Total GC content (%)	Gene Number (unique)			
	Genome	LSC	SSC	IR		Total	PCGs	rRNA	tRNA
*A. neglectus*	156,787	85,021	18,652	26,557	37.6	114	80	4	30
*A. officinalis*	156,786	85,030	18,650	26,553	37.6	114	80	4	30
*A. longiflorus*	156,735	85,036	18,643	26,528	37.6	114	80	4	30
*A. oligoclonos*	156,732	85,034	18,642	26,528	37.6	114	80	4	30
*A. gobicus*	156,757	85,066	18,635	26,528	37.6	114	80	4	30
*A. dauricus*	156,763	85,074	18,633	26,528	37.6	114	80	4	30
*A. brachyphyllus*	156,779	85,054	18,633	26,546	37.6	114	80	4	30
*A. angulofractus*	156,744	85,004	18,640	26,550	37.6	114	80	4	30
*A. schoberioides*	156,875	85,120	18,643	26,556	37.6	114	80	4	30
*A. filicinus*	156,998	85,154	18,732	26,556	37.5	114	80	4	30
*tibeticus*	156,683	85,099	18,702	26,441	37.5	114	80	4	30
*A. cochinchinensis*	156,349	84,559	18,684	26,553	37.5	114	80	4	30
*A. taliensis*	155,948	84,196	18,636	26,558	37.6	114	80	4	30
*A. myriacanthus*	156,400	84,657	18,627	26,558	37.6	114	80	4	30
*A. subscandens*	156,985	85,201	18,674	26,555	37.5	114	80	4	30
*A. munitus*	156,381	84,636	18,627	26,559	37.6	114	80	4	30
*A. meioclados*	156,535	84,798	18,645	26,546	37.5	114	80	4	30
*A. lycopodineus*	156,538	84,866	18,638	26,517	37.5	114	80	4	30
*A. trichoclados*	156,828	85,029	18,667	26,566	37.5	114	80	4	30
*A. falcatus*	157,128	85,338	18,676	26,557	37.5	114	80	4	30
*A. setaceus*	156,997	85,343	18,652	26,501	37.5	114	80	4	30
*A. virgatus*	157,064	85,319	18,697	26,524	37.5	114	80	4	30
*A. aethiopicus*	157,069	85,246	18,677	26,573	37.5	114	80	4	30
*A. densiflorus*	157,095	85,306	18,677	26,556	37.5	114	80	4	30
*A. macowanii*	156,968	85,233	18,621	26,557	37.5	114	80	4	30

### Characterization of SSRs

**Figure 2:**
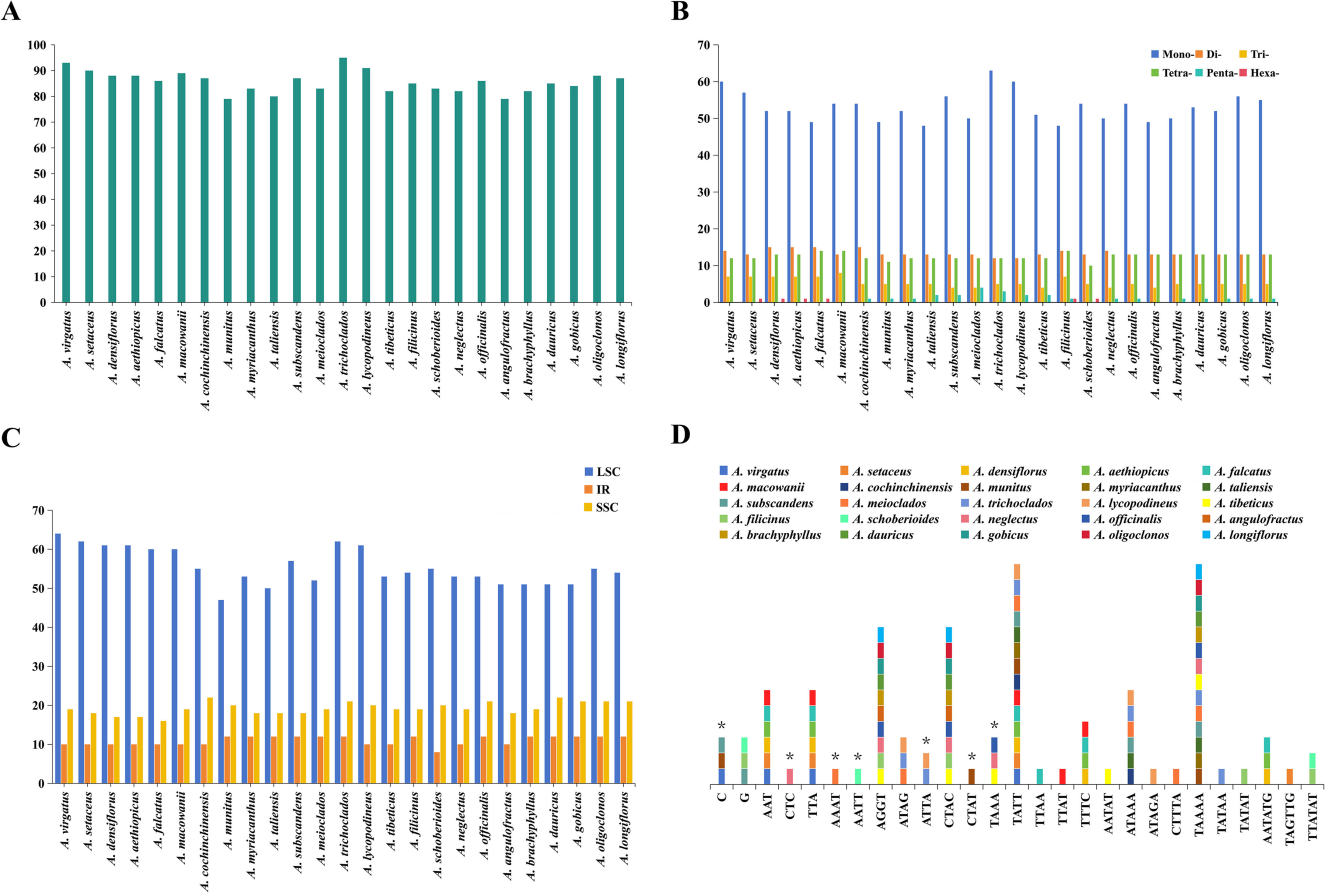
Analyses of simple sequence repeats (SSRs) in 25 *Asparagus* plastomes: **(A)** numbers of SSRs detected in each plastome; **(B)** numbers of different repeat types; **(C)** presence of SSRs in LSC, SSC, and IR; **(D)** the unique presence or absence of SSR motifs in one or several *Asparagus* species. The asterisk indicates the absence of SSR motifs.

A total of 2,142 SSRs were identified across the 25 *Asparagus* plastomes (**Figure 2**; **Supplementary Table 5**), with the number of SSRs per species ranging from 79 (*A. munitus*, *A. angulofractus* Iljin) to 95 (*A. trichoclados* (F.T.Wang & Tang) F.T.Wang & S.C.Chen), as illustrated in **Figure 2A**. All the *Asparagus* plastomes contained mono-, di-, tri-, and tetra-nucleotide repeats; however, penta-, and hexa-nucleotide repeats varied among different *Asparagus* species (**Figure 2B**). Specifically, penta-, and hexa-nucleotide repeats were absent in *A. virgatus* Baker, *A. macowanii* Baker, and *A. angulofractus*, while both of them were present in *A. filicinus*. In addition, hexa-nucleotide repeats could be observed in *A. setaceus*, *A. densiflorus* (Kunth) Jessop, *A. aethiopicus* L., *A. falcatus*, and *A. schoberioides* Kunth, and the penta-nucleotide repeats were only found in the remaining species. Among these SSRs, mono-nucleotide repeat type was the most dominant composition (48–63), followed by di-nucleotide repeats (12–15), tetra-nucleotide repeats (10–14), and tri-nucleotide repeats (4–8). Meanwhile, the A and T repeats were the majority of SSRs in each plastome (**Supplementary Table 5**). Concerning the distribution of SSRs, the majority were located in the LSC region across all *Asparagus* plastomes (**Figure 2C**).

To further investigate the differences between SSRs, 27 of 41 kinds of SSR motifs with unique presence or absence in one or several plastomes were ultimately recognized after excluding the shared repeat sequences (**Figure 2D**; **Supplementary Table 5**). Among these, the tetra-nucleotide repeats constituted the main component, followed by penta-nucleotide repeats. Notably, C repeat sequences were only absent in *A. virgatus*, *A. munitus*, and *A. subscandens*, while the G repeat sequences were exclusively found in *A. subscandens*, *A. filicinus*, and *A. schoberioides*. In addition, CTC, AAAT, AATT, and CTAT repeat sequences were absent in *A. neglectus* Kar. & Kir., *A. meioclados*, *A. schoberioides*, and *A. munitus*, respectively; TTAA, TTAT, AATAT, ATAGA, CTTTA, TATAA, TATAT, TAGTTG repeat sequences were detected solely in *A. falcatus*, *A. macowanii*, *A. tibeticus* F.T.Wang & S.C.Chen, *A. lycopodineus*, *A. meioclados*, *A. trichoclados*, *A. filicinus*, and *A. setaceus*, respectively. Apart from that, AAT/TTA repeat sequences were identified in *A.setaceus*, *A. virgatus*, *A. macowanii*, *A. falcatus*, *A. densiflorus*, and *A. aethiopicus.* CTAC/AGGT repeats were detected in *A. filicinus*, *A. tibeticus*, *A. officinalis*, *A. neglectus*, *A. angulofractus*, *A. brachyphyllus*, *A. gobicus*, *A. dauricus* Link, *A. longiflorus* Franch., and *A. oligoclonos* Maxim. The presence or absence of the remaining SSR motifs exhibited variability among *Asparagus* species.

### Plastome comparison and hotspot identification

**Figure 3:**
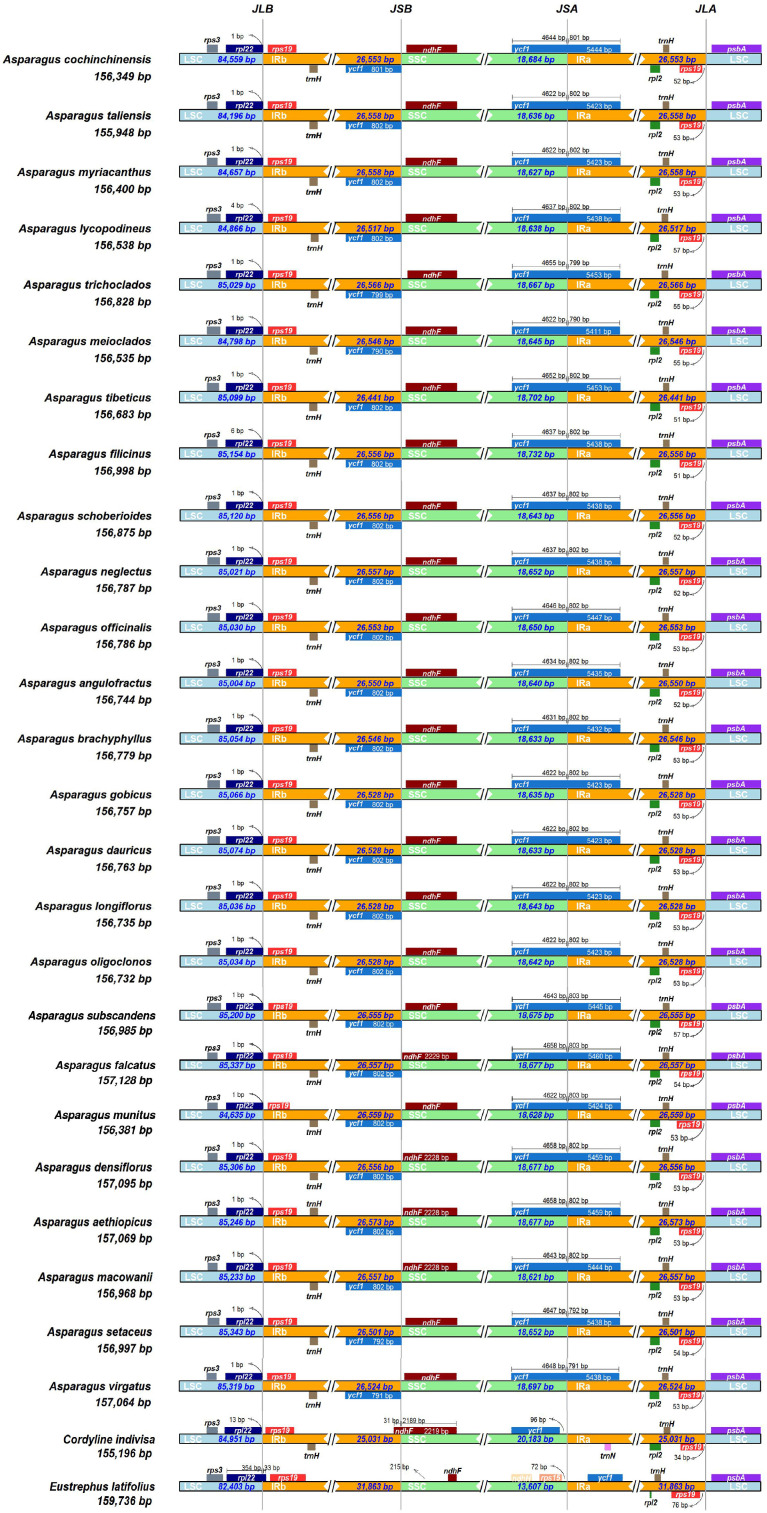
Comparison of the borders of the LSC, SSC, and IR regions among 25 *Asparagus* plastomes. JLB, junction line between LSC and IRb; JSB, junction line between IRb and SSC; JSA, junction line between SSC and IRa; JLA, junction line between IRa and LSC. These features are not to scale.

**Figure 4:**
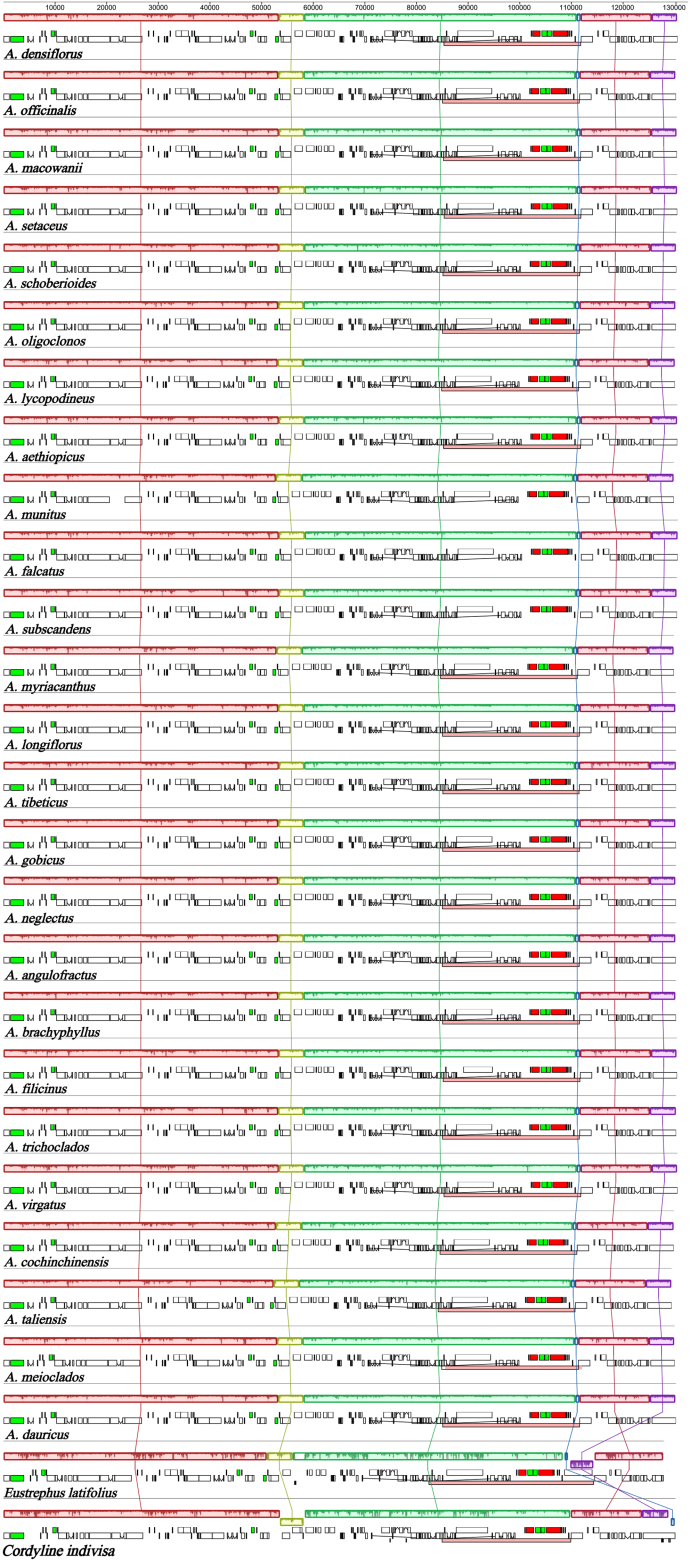
Mauve alignment of plastomes from 25 *Asparagus* species and outgroups. Rectangular blocks of the same color indicate collinear regions of sequences and the histograms within each block indicate the degree of sequence similarity.

The expansion and contraction of the IRs were compared among the *Asparagus* species and the outgroups. Overall, no significant variation of IR length was detected within *Asparagus* plastomes. The boundaries of the LSC/IRb, IRb/SSC, SSC/IRa, and IRa/LSC are relatively conserved (**Figure 3**). Specifically, the LSC/IRb junctions were located between *rpl22* and *rps19*; the boundaries of IRb/SSC were situated downstream from the *ndhF* but fell into *ycf1* (the partially duplicated gene); the SSC/IRa borders were crossed by the *ycf1*; the junctions of IRa/LSC were positioned between *rps19* and *psbA*. Furthermore, conservation in gene arrangement among *Asparagus* plastomes was confirmed with no evidence of gene rearrangement detected (**Figure 4**).

**Figure 5:**
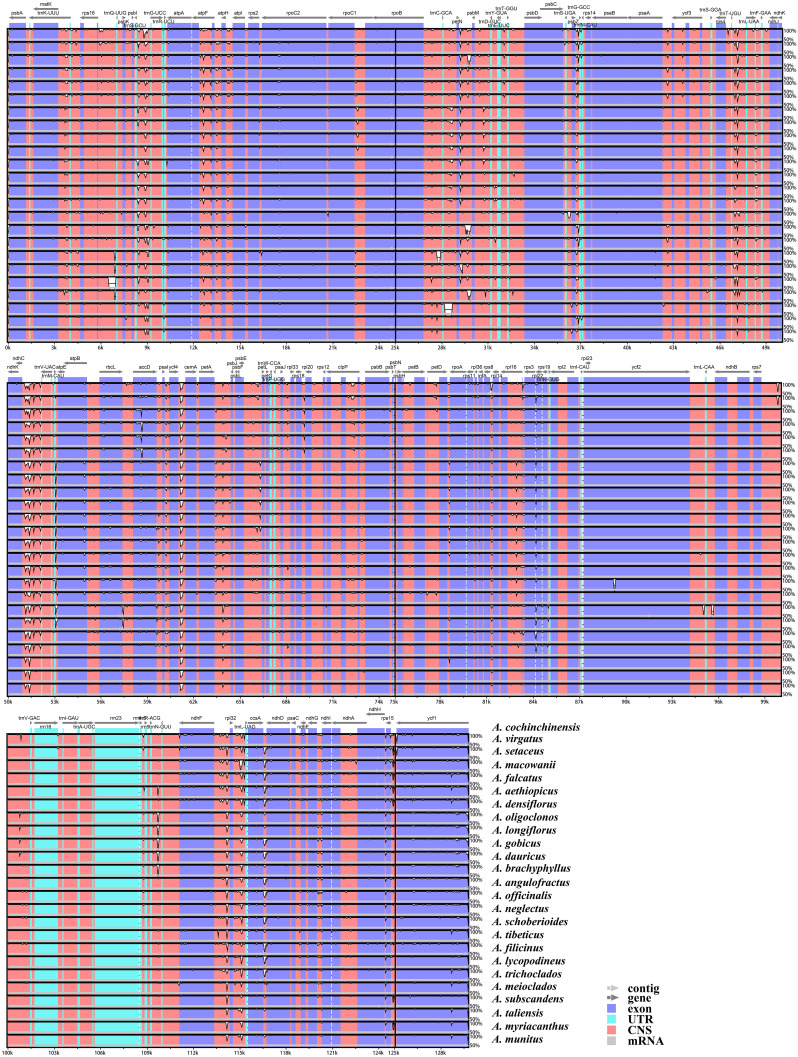
mVISTA visualization of the alignment for the 25 *Asparagus* plastomes.

**Figure 6:**
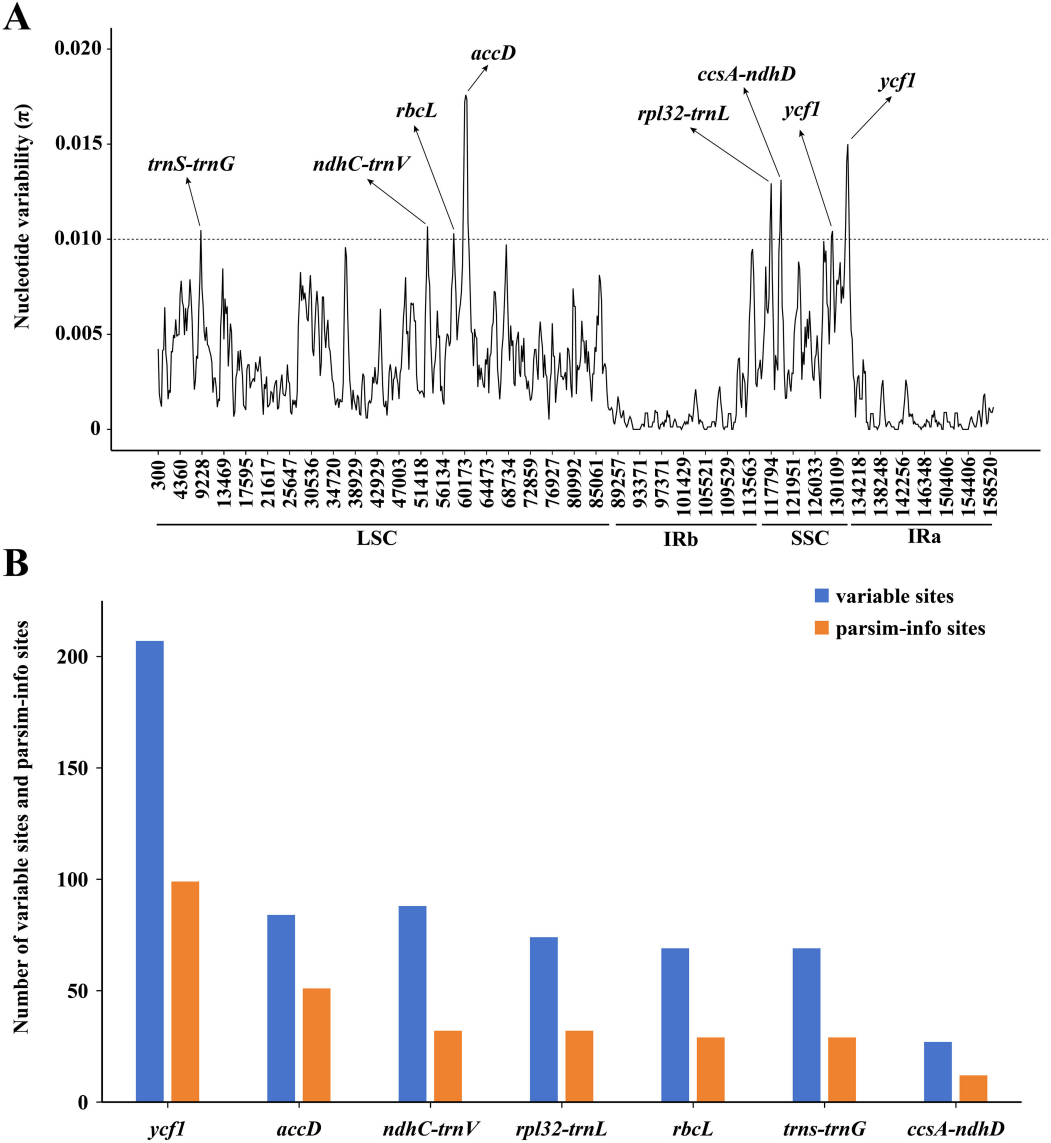
**(A)** Sliding window analysis of the 25 *Asparagus* plastomes (window length: 600 bp, step size: 200 bp) to show high variable regions (HVRs). X-axis: position of the midpoint of a window; Y-axis: nucleotide variability values (π) of each window. The dashed line indicates that the π value is equal to 0.010; **(B)** Number of variable sites and parsim-info sites in the alignment of divergence hotspots.

The results of the mVISTA analysis indicated that the alignment of *Asparagus* plastomes exhibited high similarity in the sequence identity plots (**Figure 5**). Nonetheless, certain sequence variations among different *Asparagus* plastomes were still evident. Overall, the divergence levels in noncoding regions were much higher compared to those in coding regions. Among these, three coding regions (*rbcL*, *accD*, and *ycf1*) and 12 non-coding regions (*trnS-trnG*, *rpoB-trnC*, *trnC-petN*, petN-psbM, *trnT-trnL*, *ndhC-trnV*, *petA-psbJ*, *psbE-petL*, *rpl16* intron, *ndhF-rpl32*, *rpl32-trnL* and *ccsA-ndhD*) displayed relatively significant difference. In the sliding-window analysis, the LSC and SSC regions exhibited greater nucleotide diversity with more peaks compared to the two IR regions (**Figure 6A**). Ultimately, seven regions—*accD*, *rbcL*, *ycf*1, *rpl32*-*trnL*, *ccsA*-*ndhD*, *trnS*-*trnG*, and *ndhC-trnV*—were identified as divergence hotspots with nucleotide variability values (π) ≥ 0.010. Among these, *ycf1* contained the most variable sites, followed by *ndhC-trnV*, whereas *ccsA*-*ndhD* had the fewest. Additionally, *ycf1* displayed the most abundance of parsim-info sites, followed by *accD*; however, *ccsA-ndhD* exhibited the smallest number of parsimony-informative (parsim-info) sites (**Figure 6B**; **Supplementary Table 6**).

### Phylogenetic analyses

**Figure 7:**
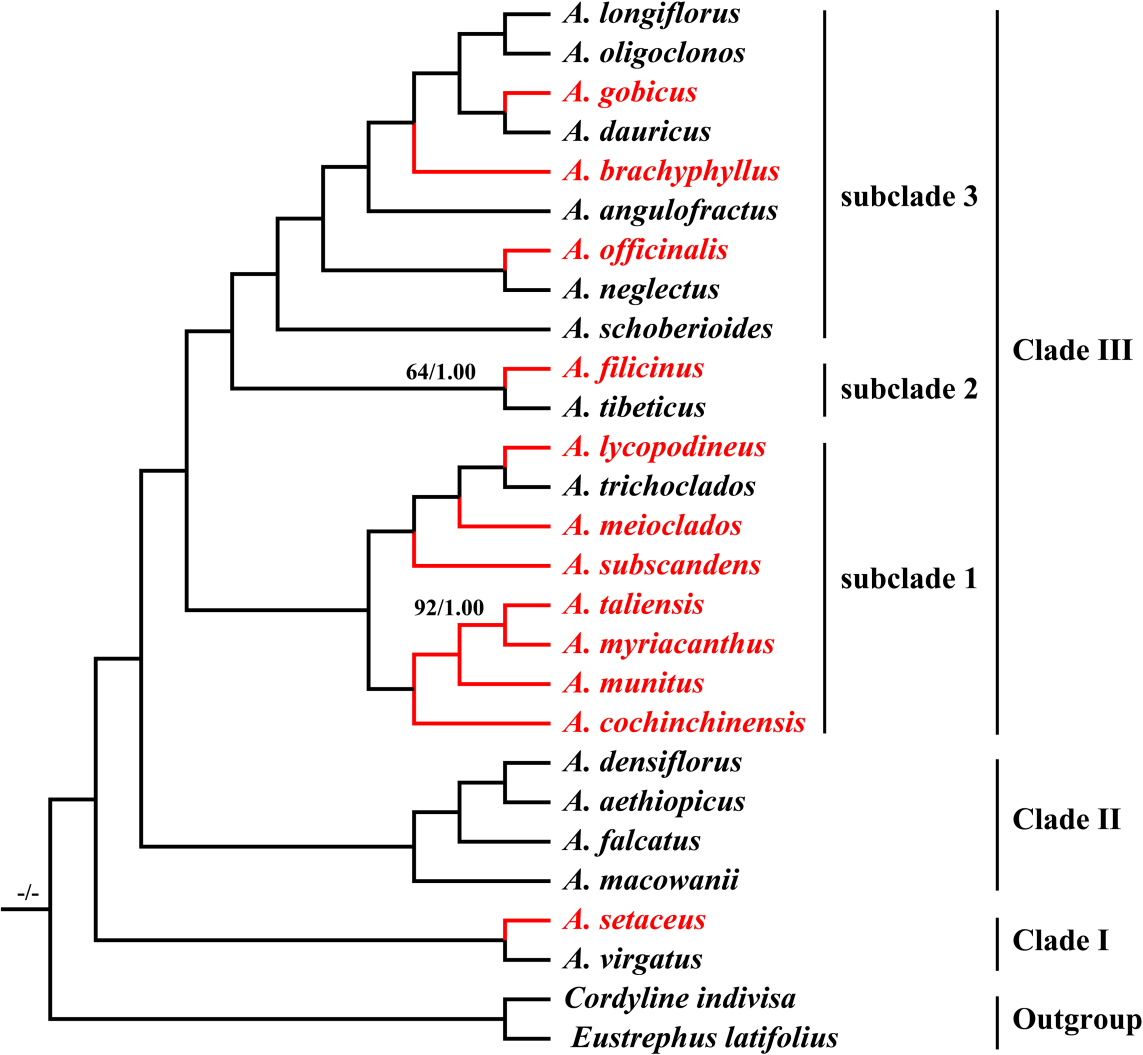
Phylogeny of the 25 *Asparagus* species inferred from maximum likelihood (ML) and Bayesian (BI) analyses based on the complete plastomes. The values above the nodes represent the ML bootstrap values (BS) and the posterior probabilities values (PP) and are shown only on branches with < 99% support. The highlighted red color represents the medicinal *Asparagus* species.

The final alignment was 176,103 bp in length, in which we identified 18,001 variable sites (10.22%) with 4,139 (2.35%) being parsimoniously informative. Both ML and BI analyses yielded identical tree topologies with strong support (Bootstrap values (BS) = 100 and posterior probability (PP) = 1.00) (**Figure 7**). All *Asparagus* species were strongly supported as monophyletic (BS = 100; PP = 1.00) and resolved into three clades: (I) *A.setaceus* was clustered with *A. virgatus* (BS = 100; PP = 1.00) at the base of the phylogenetic tree; (II) *A. macowanii* and *A. falcatus* were successively sister to the clade comprising *A. densiflorus* and *A. aethiopicus* (BS = 100; PP = 1.00); (III) the remaining species formed a distinct clade (BS = 100; PP = 1.00), which was further divided into subclade 1, 2, and 3. Within subclade 1, *A. cochinchinensis* and *A. myriacanthus* were sequentially sister to the *A. munitus* + *A. taliensis* clade; while *A. subscandens* and *A. meioclados* were successively sister to the *A. lycopodineus* + *A. trichoclados* clade. Additionally, *A. filicinus* and *A. tibeticus* formed the subclade 2 (BS = 74; PP = 1.00) and were sister to the subclade 3, which included *A. schoberioides*, *A. officinalis*, *A. neglectus*, *A. angulofractus*, *A. brachyphyllus*, *A. gobicus*, *A. dauricus*, *A. longiflorus*, and *A. oligoclonos* (BS = 100; PP = 1.00). In subclade 3, *A. schoberioides* was the first to diverge from the remainders, followed by the *A. officinalis* + *A. neglectus* clade. Subsequently, *A. angulofractus* and *A. schoberioide* were successively sister to a clade in which the *A. gobicus* + *A. dauricus* clade was clustered with the *A. longiflorus* + *A. oligoclonos* clade.

## Discussion

### Chloroplast genome structure

In this study, we conducted the sequencing, assembly, and annotation of 21 plastomes from *Asparagus* species and compared them with other four *Asparagus* plastomes. All *Asparagus* plastomes exhibited a typical quadripartite molecular structure consistent with those in Asparagaceae (Lu et al., 2022; Sheng, 2022; Van et al., 2022; Wang et al., 2022b; Wong et al., 2022; Huang et al., 2023; Tian et al., 2023; Wang et al., 2023). The pattern for the boundaries of the LSC/IRb, IRb/SSC, SSC/IRa, and IRa/LSC regions was also shared by all *Asparagus* plastomes, which was previously reported within Asparagaceae plastomes (Lu et al., 2022; Wang et al., 2022b; Huang et al., 2023). Furthermore, genome size, GC content, gene content, and order among *Asparagus* plastomes were highly similar. These phenomena indicated that the plastomes of *Asparagus* were highly conservative. Notably, the pseudogenization of the *ycf*15 gene, frequently documented across a wide range of lineages (Dong et al., 2013; Shi et al., 2013; Jiao et al., 2022), was found in all *Asparagus* plastomes, which may be the synapomorphy of the genus.

### Simple sequence repeat and hotspot identification

In recent years, plastome-derived SSRs have been deemed a valuable resource for species authentication, clarifying evolutionary relationships, and assessing genetic diversity within plant populations (Provan et al., 2001; Ahmad et al., 2018; Zhou et al., 2023; Tu et al., 2024). Our study identified 79 to 95 SSRs in each *Asparagus* plastome, and types of SSRs, as well as most detected SSR motifs were also reported by previous studies (Sheng, 2022; Wong et al., 2022; Tian et al., 2023). On the whole, SSRs exhibited high levels of interspecific polymorphism across *Asparagu*s plastomes. Firstly, the quantity of SSRs of the same type varied across different *Asparagu*s species, especially for mono-nucleotide repeats. Secondly, there are differences in the composition of SSRs among *Asparagu*s plastomes. Pentanucleotide repeats were found only in Clade III species, but not in all, while hexa-nucleotide repeats were relatively dispersed in Clade I to Clade III species. Moreover, even if the types of SSRs are the same, the specific SSR motifs in *Asparagu*s plastomes were diverse (**Supplementary Table 5**). Through further comparison, a tatal of 12 special SSR motifs were found to be unique in specific species, which may serve as effective markers for species identification (**Figure 2D**).

Despite 15 regions showing significant sequence differences through mVISTA analysis, only seven regions (*accD*, *rbcL*, *ycf1*, *trnS-trnG*, *ndhC-trnV*, *rpl32-trnL*, *ccsA-ndhD*) corresponded to the results of the sliding-window analysis. Notably, the other regions exhibiting relatively high divergence yet low π values may be attributed to the presence of gaps within the compared matrix. Hence, we selected the above seven regions as candidate barcodes for *Asparagus*, with five (*accD*, *ycf1*, *trnS-trnG*, *rpl32-trnL*, and *ndhC-trnV)* reported by previous studies (Wong et al., 2022; Tian et al., 2023) and two (*rbcL* and *ccsA-ndhD*) firstly determined in this study. Noteworthy, despite exceeding 2,000 bp in length, the *ycf1* sequence has been proposed as the most promising plastid DNA barcode of land plants (Dong et al., 2015). The successful design and implementation of taxon-specific PCR primers spanning the large locus *ycf1* across different taxa (Neubig and Abbott, 2010; Parks et al., 2011; Dong et al., 2015) underscore its potential application as a marker for species identification. Nevertheless, the reliability of these sequences as DNA barcodes for species identification remains to be confirmed (Chao et al., 2023; Chac and Thinh, 2023) and requires more intra- and inter-species *Asparagus* sampling in future studies (Zhang et al., 2010; Gao et al., 2012).

### Phylogeny inference

Although ITS and several plastome fragments have been widely utilized for the phylogenetic reconstruction of *Asparagus*, studies employing these data often produced phylogenetic trees with weak support or low resolution (Fukuda et al., 2005; Ou et al., 2010; Kubota et al., 2012; Ou et al., 2013). In this study, we performed phylogenetic analyses based on plastome data. As expected, the robust phylogenetic framework for *Asparagus* taxa was yielded, with high support values for nearly all nodes. All 25 *Asparagus* species were divided into three clades and the phylogenetic positions of most *Asparagus* species were largely congruent with findings from earlier researches (Kubota et al., 2012; Norup et al., 2015; Bentz et al., 2024). Apparently, medicinal *Asparagus* species were mainly distributed in Clade III, especially the subclade 1 (**Figure 7**). Notably, the close relationships between *A. cochinchinensis*, *A. munitus*, *A. taliensis*, and *A. myriacanthus* were identified, which was different from those of Wong et al. (2022) and Tian et al. (2023) but basically consistent with that of Bentz et al. (2024). Their affinities were further supported by similar morphological evidence—typically 3-angled cladodes, spinescent leaf spurs, and main stems with sharp spines. We also revealed the affinity between *A. subscandens* and the previously reported group including *A. meioclados*, *A. lycopodineus*, and *A. trichoclados* (Bentz et al., 2024). Similar leaf spur characteristics—either not spinescent or indistinctly spinescent—may justify their phylogenetic location found here. Additionally, it is worth noting that the swollen roots of many species in subclade 1, such as *A. munitus*, *A. subscandens*, *A. lycopodineus*, *A. meioclados* and *A. taliensis*, on the whole, have some similar medicinal value with that of *A. cochinchinensis* (Editorial Committee of Chinese Materia Medica of the National Administration of Traditional Chinese Medicine, 1999; Yunnan Food and Drug Administration, 2005; Sichuan Food and Drug Administration, 2010; Lv and Chen, 2019). We also identified a strong affinity between *A. gobicus* and *A. dauricus* (BS = 100; PP = 1.00). However, this finding was inconsistent with that of Bentz et al. (2024), who determined *A. gobicus* was closely related to *A. kiusianus*, a species endemic to Japan, and found *A. dauricus* to be sister to the *A. breslerianus* + *A. longiflorus* clade. These discrepancies may arise from differences in taxonomic sampling and molecular variations, highlighting the need for further investigation into their phylogenetic relationships. Overall, based on the robust phylogenetic framework, the relationships among the Chinese group of medicinal species and allied taxa within *Asparagus* were preliminarily elucidated.

## Conclusion

In this study, we conducted more comprehensive analyses of the characteristics and structural variations of *Asparagus* plastomes than previously. These results indicate that the *Asparagus* plastomes are relatively conserved in terms of the structure, genome size, and gene content. Nevertheless, the quantities and types of SSRs exhibit high levels of interspecific polymorphism within *Asparagus.* The special SSR motifs and selected divergent hotspot regions from *Asparagus* plastomes may serve as potential molecular markers for future research on interspecific diversity and the identification of *Asparagus*. Our robust plastome-based phylogeny has provided preliminary insights into phylogenetic relationships among the Chinese group of medicinal species and related taxa within *Asparagus*. In conclusion, this study offers a wealth of informative genetic resources pertinent to *Asparagus*, significantly enhancing our comprehension of the evolution of this genus and laying a foundation for its identification, assessment of genetic population diversity, exploration and conservation of germplasm resources.

## Data Availability

The datasets presented in this study can be found in online repositories. The names of the repository/repositories and accession number(s) can be found in the article/**Supplementary Material.**
