# Supplementary material for: Plastid genome comparison and phylogenetic analyses of the Chinese group of medicinal species and related taxa within Asparagus genus
Source: Front Plant Sci. 2025 Jan 27;16:1508898. doi: 10.3389/fpls.2025.1508898 (PMC11808011; doi:10.3389/fpls.2025.1508898)
Supplement: Supplementary file 1 [file Table1.docx]

**Supplementary Table 1 21 species newly sequenced with voucher and GenBank accession numbers**

| **Samples** | **Locality** | **Isolate** | **Voucher** | **GenBank accession** |
| --- | --- | --- | --- | --- |
| *Asparagus cochinchinensis* | Guangzhou, Guangdong | XPX-2 | XPX-2 | QQ851333 |
| *Asparagus taliensis* | Kunming, Yunnan | XPX-6 | XPX-6 | QQ851339 |
| *Asparagus myriacanthus* | Garze, Sichuan | S1309 | SCSB-B-000454 | QQ851319 |
| *Asparagus lycopodineus* | Daguan, Yunnan | JYH462 | 2019145 | QQ851326 |
| *Asparagus trichoclados* | Gaoligong Mountain, Yunnan | S12827 | GLGE12287 | QQ851314 |
| *Asparagus meioclados* | Kunming, Yunnan | XPX-16 | XPX-16 | QQ851343 |
| *Asparagus filicinus* | Qujing, Yunnan | S11629 | 10CS1940 | QQ851309 |
| *Asparagus tibeticus* | Lhasa, Tibet | S1315 | ZhongY130 | QQ851321 |
| *Asparagus schoberioides* | Panshi, Jilin | JYH437 | AHC2019001 | QQ851328 |
| *Asparagus neglectus* | Fuyun, Xinjiang | S09336 | 16CS13513 | QQ851311 |
| *Asparagus officinalis* | Kunming, Yunnan | XPX-13 | XPX-13 | QQ851342 |
| *Asparagus angulofractus* | Hotan, Xinjiang | S10200 | 08CS299 | QQ851310 |
| *Asparagus brachyphyllus* | Qamdo, Tibet | S11014 | Xianh0366 | QQ851320 |
| *Asparagus gobicus* | Xilingol, Inner Mongolia | S9335 | NMZA0196 | QQ851318 |
| *Asparagus dauricus* | Kunming, Yunnan | XPX-12 | XPX-12 | QQ851330 |
| *Asparagus longiflorus* | Haidong, Qinghai | S1306 | ChenSL1751 | QQ851313 |
| *Asparagus oligoclonos* | Panshi, Jilin | JYH440 | AHC2019004 | QQ851317 |
| *Asparagus densiflorus* | Kunming, Yunnan | JYH007 | 2019078 | QQ851322 |
| *Asparagus macowanii* | Kunming, Yunnan | JYH026 | 2019099 | QQ851347 |
| *Asparagus setaceus* | Kunming, Yunnan | JYH029 | 2019103 | QQ851331 |
| *Asparagus virgatus* | Guangzhou, Guangdong | XPX-1 | XPX-1 | QQ851332 |
