# Supplementary material for: Plastid genome comparison and phylogenetic analyses of the Chinese group of medicinal species and related taxa within Asparagus genus
Source: Front Plant Sci. 2025 Jan 27;16:1508898. doi: 10.3389/fpls.2025.1508898 (PMC11808011; doi:10.3389/fpls.2025.1508898)
Supplement: Supplementary file 2 [file Table2.docx]

**Supplementary Table 2 Plastomes downloaded from GenBank**

| **Species** | **Family** | **GenBank accession** |
| --- | --- | --- |
| *Asparagus aethiopicus* | Asparagaceae | MZ337394 |
| *Asparagus falcatus* | Asparagaceae | PP175306 |
| *Asparagus munitus* | Asparagaceae | OQ628361 |
| *Asparagus subscandens* | Asparagaceae | PP180014 |
| *Cordyline indivisa* | Asparagaceae | KX822776 |
| *Eustrephus latifolius* | Asparagaceae | KM233639 |
