# Supplementary material for: Plastid genome comparison and phylogenetic analyses of the Chinese group of medicinal species and related taxa within Asparagus genus
Source: Front Plant Sci. 2025 Jan 27;16:1508898. doi: 10.3389/fpls.2025.1508898 (PMC11808011; doi:10.3389/fpls.2025.1508898)
Supplement: Supplementary file 3 [file Table3.docx]

**Supplementary Table 3 Summary of Illumina sequencing for plastomes of 21 samples**

| **Voucher** | **Species** | **No. of total**  **reads** | **Size of chloroplast genome (bp)** | **No. of mapped reads** | **Coverage (×)** |
| --- | --- | --- | --- | --- | --- |
| 08CS299 | 1. *angulofractus* | 38,832,514 | 156,744 | 1,748,750 | 1,606.569 |
| 16CS13513 | 1. *neglectus* | 19,707,834 | 156,787 | 1,697,076 | 1631.168 |
| ChenSL1751 | *A. longiflorus* | 18,190,284 | 156,735 | 1,314,730 | 1,266.623 |
| GLGE12287 | *A. trichoclados* | 17,055,504 | 156,828 | 404,057 | 389.042 |
| AHC2019004 | *A. oligoclonos* | 20,126,940 | 156,732 | 1,391,241 | 1,340.361 |
| NMZA0196 | *A. gobicus* | 21,814,832 | 156,757 | 1,131,068 | 1,081.777 |
| SCSB-B-000454 | *A. myriacanthus* | 19,697,468 | 156,400 | 1,400,508 | 1,352.153 |
| XiaNH0366 | *A. brachyphyllus* | 8,818,552 | 156,779 | 524,153 | 481.429 |
| ZhongY130 | *A. tibeticus* | 25,011,054 | 156,683 | 1,859,192 | 1,791.758 |
| 10CS1940 | *A. filicinus* | 8,200,612 | 156,998 | 393,546 | 360.964 |
| 2019078 | *A. densiflorus* | 23,075,420 | 157,095 | 937,955 | 636.548 |
| 2019145 | *A. lycopodineus* | 24,059,446 | 156,538 | 897,850 | 857.175 |
| AHC2019001 | *A. schoberioides* | 19,409,302 | 156,875 | 769,409 | 733.175 |
| XPX-12 | *A. dauricus* | 34,355,492 | 156,763 | 2,497,687 | 2,405.366 |
| 2019103 | *A. setaceus* | 20,024,784 | 156,997 | 560,876 | 444.949 |
| XPX-1 | *A. virgatus* | 28,946,506 | 157,064 | 938,084 | 901.572 |
| XPX-2 | *A. cochinchinensis* | 28,288,868 | 156,349 | 736,605 | 711.087 |
| XPX-6 | *A. taliensis* | 26,896,650 | 155,948 | 1,710,091 | 1,652.539 |
| XPX-13 | *A. officinalis* | 31,860,884 | 156,786 | 1,478,090 | 1,423.175 |
| XPX-16 | *A. meioclados* | 29,222,434 | 156,535 | 743,047 | 714.249 |
| 2019099 | *A. macowanii* | 32,055,902 | 156,986 | 376,077 | 361.719 |
