# Supplementary material for: Plastid genome comparison and phylogenetic analyses of the Chinese group of medicinal species and related taxa within Asparagus genus
Source: Front Plant Sci. 2025 Jan 27;16:1508898. doi: 10.3389/fpls.2025.1508898 (PMC11808011; doi:10.3389/fpls.2025.1508898)
Supplement: Supplementary file 6 [file Table6.docx]

**Supplementary Table 6 Characteristics of alignment of divergence hotspots in *Asparagus* plastomes**

| **Divergence hotspots** | **Length** | **Number of variable sites** | **Number of parsim-info sites** |
| --- | --- | --- | --- |
| ***accD*** | 1,589 | 84 | 51 |
| ***rbcL*** | 1,348 | 69 | 29 |
| ***ycf1*** | 5,472 | 207 | 99 |
| ***ccsA-ndhD*** | 259 | 27 | 12 |
| ***ndhC-trnV*** | 1,971 | 88 | 32 |
| ***rpl32-trnL*** | 1,048 | 74 | 32 |
| ***trnS-trnG*** | 1,348 | 69 | 29 |
